# Secondary-type mutations do not impact outcome in *NPM1*-mutated acute myeloid leukemia – implications for the European LeukemiaNet risk classification

**DOI:** 10.1038/s41375-023-02016-6

**Published:** 2023-09-07

**Authors:** Jan-Niklas Eckardt, Marius Bill, Christian Rausch, Klaus Metzeler, Karsten Spiekermann, Sebastian Stasik, Tim Sauer, Sebastian Scholl, Andreas Hochhaus, Martina Crysandt, Tim H. Brümmendorf, Utz Krug, Bernhard Wörmann, Wolfgang Hiddemann, Dennis Görlich, Cristina Sauerland, Björn Steffen, Hermann Einsele, Andreas Neubauer, Andreas Burchert, Kerstin Schäfer-Eckart, Wolfgang E. Berdel, Christoph Schliemann, Stefan W. Krause, Mathias Hänel, Maher Hanoun, Martin Kaufmann, Lars Fransecky, Jan Braess, Leo Ruhnke, Johannes Schetelig, Jan Moritz Middeke, Hubert Serve, Claudia D. Baldus, Uwe Platzbecker, Carsten Müller-Tidow, Martin Bornhäuser, Tobias Herold, Christian Thiede, Christoph Röllig

**Affiliations:** 1 Department of Internal Medicine I, University Hospital Carl Gustav Carus, Technical University Dresden, Dresden, Germany; 2 Mildred Scheel Early Career Center, Medical Clinic and Policlinic I, University Hospital of the Technical University Dresden, Dresden, Germany; 3 National Center for Tumor Diseases Dresden (NCT/UCC), Medical Faculty and University Hospital Carl Gustav Carus, Technical University Dresden, Dresden, Germany; 4 German Cancer Consortium (DKTK), Dresden, Germany; 5 Laboratory for Leukemia Diagnostics, Department of Medicine III, LMU University Hospital, LMU Munich, Munich, Germany; 6 Medical Clinic and Policlinic I Hematology and Cell Therapy, University Hospital, Leipzig, Germany; 7 German Cancer Research Center (DKFZ) and Medical Clinic V, University Hospital Heidelberg, Heidelberg, Germany; 8 Klinik für Innere Medizin II, Jena University Hospital, Jena, Germany; 9 Department of Hematology, Oncology, Hemostaseology, and Cell Therapy, University Hospital RWTH Aachen, Aachen, Germany; 10 Department of Medicine III, Hospital Leverkusen, Leverkusen, Germany; 11 Department of Hematology, Oncology and Tumor Immunology, Charité, Berlin, Germany; 12 Institute for Biostatistics and Clinical Research, University Muenster, Muenster, Germany; 13 Medical Clinic II, University Hospital Frankfurt, Frankfurt (Main), Germany; 14 Medical Clinic and Policlinic II, University Hospital Würzburg, Würzburg, Germany; 15 Department of Hematology, Oncology and Immunology, Philipps-University-Marburg, Marburg, Germany; 16 Department of Internal Medicine V, Paracelsus Medizinische Privatuniversität and University Hospital Nuremberg, Nuremberg, Germany; 17 Department of Medicine A, University Hospital Münster, Münster, Germany; 18 University Hospital Erlangen, Erlangen,, Germany; 19 Medical Clinic III, Chemnitz Hospital AG, Chemnitz, Germany; 20 Department of Hematology, University Hospital Essen, Essen, Germany; 21 Department of Hematology, Oncology and Palliative Care, Robert-Bosch-Hospital, Stuttgart, Germany; 22 Department of Internal Medicine, University Hospital Kiel, Kiel, Germany; 23 Hospital Barmherzige Brueder Regensburg, Regensburg, Germany

**Keywords:** Acute myeloid leukaemia, Translational research

## To the Editor:

In 2022, the European LeukemiaNet (ELN) risk classification for Acute Myeloid Leukemia (AML) was updated for the second time [1]. Since the first edition in 2010 (ref. [2]), it has become one of the most commonly used systems to assess the prognosis of AML patients and to guide therapeutic decisions. A major novelty of ELN2022 is that “secondary-type” mutations (STM, also called myelodysplasia-related gene mutations), i.e., mutations in the genes, *SRSF2*, *SF3B1*, *U2AF1*, *ZRSR2*, *ASXL1*, *EZH2*, *BCOR*, and *STAG2*, were deemed adverse risk. The importance of STMs is further emphasized by the novel 5th edition of the World Health Organization (WHO) classification of myeloid neoplasms [3] and the International Consensus Classification (ICC) of Myeloid Neoplasms and Acute Leukemias [4]. Patients harboring STMs are categorized as “AML, myelodysplasia-related” (AML-MR, WHO) and “AML with myelodysplasia-related gene mutations” (ICC), respectively, regardless of additional cytogenetic aberrations or a medical history of hematological malignancies. The decision to include STMs as prognostic markers was based on studies showing poor prognosis also in de novo AML patients [5, 6]. However, a pertinent question also raised by the ELN is whether STMs abrogate the positive prognostic value of co-occurring favorable markers, especially *NPM1* mutations. For the time being, these patients are classified as favorable in ELN2022 with STMs not being considered adverse risk in that context.

To address the question if this assumption is valid, we investigated a pooled cohort of 936 *NPM1*-mutated AML patients who were treated in previously reported multicenter trials of the Study Alliance Leukemia (SAL; AML96 [NCT00180115], AML2003 [NCT00180102], AML60+ [NCT00180167], SORAML [NCT00893373]) and the AML Cooperative Group (AMLCG-1999 [NCT00266136], AMLCG2008 [NCT01382147]). All patients in these previously conducted trials were treated with intensive induction chemotherapy. Trial protocols are summarized in Table S1. Eligibility was determined based on diagnosis of non-APL AML, age ≥18 years, and *NPM1* mutation detected in targeted sequencing. All patients gave their written informed consent according to the revised Declaration of Helsinki [7]. All studies were approved by all local Institutional Review Boards. Complete remission (CR), relapse-free (RFS), and overall survival (OS) were defined according to ELN2022 [1]. Patients were retrospectively re-stratified according ELN2022 risk categories [1]. Standard techniques for chromosome banding and fluorescence-in-situ-hybridization (FISH) were used for karyotyping. For the SAL cohort, next-generation sequencing (NGS) was performed using the TruSight Myeloid Sequencing Panel (Illumina, San Diego, CA, USA). Pooled samples were sequenced paired-end and a 5% variant allele frequency (VAF) mutation calling cut-off was used with human genome build HG19 as a reference as previously described in detail [8]. For the AMLCG cohort, targeted gDNA sequencing of 68 genes associated with myeloid malignancies was performed using a VAF cut-off of 2% as previously reported in detail [9]. Statistical analysis was done with STATA BE 17.0 (Stata Corp, College Station, TX, USA). Statistical significance was determined using a significance level α of 0.05. All tests were carried out as two-sided tests. Normality was assessed using the Shapiro-Wilk test. For non-normal continuous data, the Wilcoxon rank sum test was used. Categorical data was assessed using Fisher’s exact test. Median follow-up time was calculated using reverse Kaplan-Meier analysis [10]. To obtain odds ratios (OR) for CR, logistic regression was used. Time-to-event analysis for RFS and OS was performed with the Kaplan-Meier-method as well as the log-rank test and Cox-proportional hazard models were employed to obtain hazard ratios (HR). 95%-confidence-intervals (95%-CI) are reported for all point estimates.

**Fig. 1:**
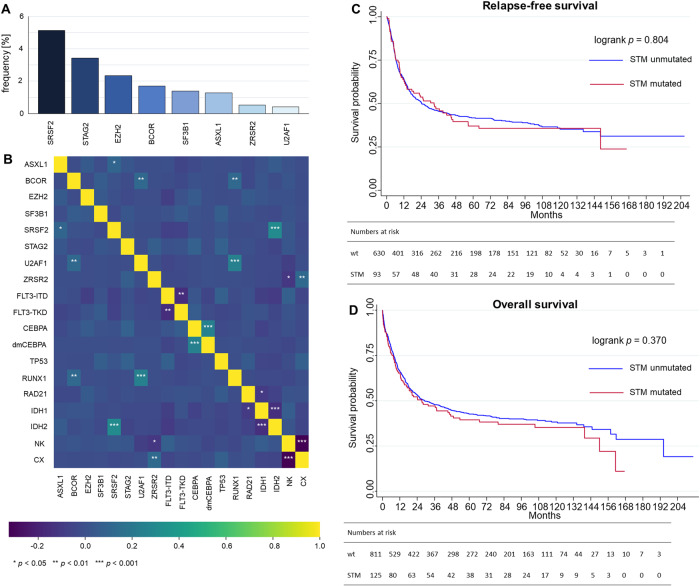
Distribution, co-mutational landscape and impact on outcome of secondary-type mutations in *NPM1*-mutated AML. Secondary-type mutations (STM) were present in 125 of 936 (13.4%) *NPM1*-mutated patients with alterations of *SRSF2, STAG2*, and *EZH2* being the most frequent (**A**). Correlation heatmap (**B**) using Spearman correlation coefficients of individual STMs and relevant co-occurring mutations as well as normal (NK) and complex karyotypes (CX, ≥3 aberrations). Statistical significance is indicated as asterisks on three different levels. The Benjamini-Hochberg method was used to adjust for multiple testing. Kaplan-Meier plots and corresponding log-rank tests for relapse-free survival (**C**) and overall survival (**D**) show no significant differences between *NPM1*-mutant AML patients with regard to presence or absence of STM.

In our multicenter cohort of 936 *NPM1*-mutated AML patients, we found 125 patients (13.4%) harboring at least one STM. In order from most frequent to least frequent, co-occurring STMs were *SRSF2* (*n* = 48; 5.1%), *STAG2* (*n* = 32; 3.2%), *EZH2* (*n* = 22, 2.4%), *BCOR* (*n* = 16; 1.7%), *SF3B1* (*n* = 13; 1.4%), *ASXL1* (*n* = 12; 1.3%), *ZRSR2* (*n* = 5; 0.5%), and *U2AF1* (*n* = 4; 0,4%; Fig. 1A). Patients with a STM were significantly older than STM negative patients (median 59 vs. 55 years, *p* = 0.003) while there was no difference regarding sex (male: 49.6% vs. female: 50.4%, *p* = 0.08). White blood cell count and platelet count at initial diagnosis was significantly reduced for patients with co-occurring STM (22.2*10^9^/L vs. 39.7*10^9^/L, *p* < 0.001 and 46.5*10^9^/L vs. 65.0 10^9^/L, *p* < 0.001, respectively), while hemoglobin levels and bone marrow blast counts did not differ (see Table S2 for baseline characteristics). Regarding the co-mutational landscape, the most apparent correlations were found for co-occurring alterations of *U2AF1* and *RUNX1* as well as *SRSF2* and *IDH2* (Fig. 1B). The rate of co-occurring *FLT3*-ITD did not differ significantly between patients with or without STM. There was no difference in the rate of patients harboring myelodysplasia-related cytogenetics as defined by ELN2022 (ref. [1]) between patients with STM and those without. Median follow-up for the entire cohort was 8.0 years.

There were no significant differences in CR rates between *NPM1*-mutated patients with or without additional STMs (74.4% vs. 77.7%, OR: 0.83 [95%-CI: 0.54–1.29], *p* = 0.416, Table 1). Median RFS for *NPM1*-mutated patients with STMs was 32.9 months (95%-CI: 13.0–46.0) while patients without STMs had a median RFS of 24.3 months (95%-CI: 18.7–33.3) corresponding to a HR of 1.04 (95%-CI: 0.79–1.37, *p* = 0.804, Fig. 1C, Table 1). Median OS for *NPM1*-mutated patients with or without STMs was 27.2 months (95%-CI: 14.2–49.0) and 29.1 months (95%-CI: 23.5–41.4), respectively, corresponding to a HR of 1.11 (95%-CI: 0.88–1.41, *p* = 0.370, Fig. 1D, Table 1). We subsequently excluded patients with co-occurring mutations in *TP53* or myelodysplasia-related cytogenetics, which all define ELN adverse risk. Despite this exclusion, patient outcome did not differ regarding CR rate, RFS, and OS between patients with or without STM (Table S3, Fig. S1). Additionally, outcomes of patients with *NPM1*-mutated AML and co-occurring mutations of *TP53* (Table S4, Fig. S2) as well as *NPM1*-mutated AML with co-occurring myelodysplasia-related cytogenetic changes (Table S5, Fig. S3) were evaluated. Both subgroups did not show any significant differences regarding CR rate, RFS or OS when compared to *TP53*-wildtype or patients without myelodysplasia-related cytogenetic changes, respectively. Next, we restricted our analysis to patients who are classified favorable risk according to ELN2022. Again, we found no significant outcome differences based on the STM status (Table S6, Fig. S4).

**Table 1:** Summary of patient outcome with respect to secondary type mutation status in *NPM1*-mutated AML.

Outcome	STM mut.	STM wt.	OR/HR	*p*
n/N (%)	125/936 (13.4)	811/936 (86.6)		
CR rate, *n* (%)	93/125 (74.4%)	630/811 (77.7)	0.83 [0.54–1.29]	0.416
RFS	32.9 [13.0–46.0]	24.3 [18.7–33.3]	1.04 [0.79–1.37]	0.804
OS	27.2 [15.2–49.0]	29.1 [23.5–41.4]	1.11 [0.88–1.41]	0.370

Survival times are displayed in months. Square brackets show 95%-confidence intervals.

*CR* complete remission, *HR* hazard ratio, *mut.* mutated, *n/N* number, *OR* odds ratio, *OS* overall survival, *RFS* relapse-free-survival, *wt* wild-type.

The rate of allogeneic hematopoietic stem cell transplantation (HSCT) between patients without or with STM differed significantly with regard to HSCT in first CR (STM-mutated: 7.2% vs. STM-wildtype: 15.4%, *p* = 0.01) while there was no difference for HSCT as salvage therapy (STM-mutated: 10.4% vs. STM-wildtype: 16.2%, *p* = 0.11, Table S2). Again, when we excluded patients who underwent allogeneic HSCT from analysis, there were no differences in RFS and OS between patients with or without STM (Table S7, Fig. S5).

We conducted an analysis on a multicenter cohort of 936 AML patients, all of whom had a *NPM1* mutation with 13% harboring an additional STM. Lindsley et al initially found and defined STMs because of their high specificity for secondary AML [6]. They also observed that *NPM1* mutations are predominantly associated with de novo AML and underrepresented in secondary or therapy-related AML [6]. Additionally, previous studies with smaller cohorts suggest that some of the STM genes are mutually exclusive with *NPM1* mutations [5, 11]. In our cohort, we found an intriguing overlap where *NPM1* could be co-mutated with every STM gene.

Consequently, given that *NPM1* mutations are among the most common mutations in AML and are well-established as prognostic markers [1, 2], the question of whether STMs alter the prognostic value of *NPM1* is of great clinical interest. We observed no impact on CR rates, RFS, and/or OS for *NPM1* mutated patients with or without STM, respectively. Therefore, the current suggestion of the ELN panel [1] that STM should not overrule the favorable impact of a co-occurring *NPM1* mutation is supported by our findings. A similar pattern was observed in a smaller analysis that STMs have no impact on the outcomes of *NPM1*-mutated AML patients [11]. This study did not include all STM genes and some were found in only a single patient whereas our cohort includes all STMs in at least four patients. Zhou et al. [12] report 25 (19%) of 129 *NPM1*-mutated AML patients to also harbor STMs (in line with our findings most commonly mutations of *SRSF2* and *STAG2*). Further, the authors also report no significant differences between patients with or without STM regarding CR rate, RFS, or OS [12]. Notably, their cohort also included patients, who received non-intensive treatment regimens or best supportive care only [12]. Recently, *NPM1* mutations have also been found to bear favorable outcomes even when occurring in therapy-related AML [13] while co-occurring chromosomal abnormalities have been associated with poorer outcomes [14] highlighting context-sensitive genetic heterogeneity in *NPM1*-mutated AML.

Interestingly, in our cohort, a higher proportion of patients without STMs received allogeneic HSCT in first CR, which is likely due to age differences, with younger patients predominantly found in the STM negative group. However, the overall frequency in both groups was small. Despite patients with an STM being older and receiving allogeneic HSCT significantly less frequently—factors that are associated with higher relapse rates and worse outcome—in our cohort, their outcomes were not adversely affected even despite co-occurring STMs. We also found no significant differences when we restricted the analyses to patients who were consolidated only with chemotherapy. These findings further strengthen the notion that the presence of STMs should not overrule the favorable risk associated with *NPM1* mutations.

In summary, we found STMs to have no adverse effect on the clinical outcome of *NPM1* mutated patients. As a result, these patients should still be considered ELN favorable risk.

### Supplementary information

Supplements

## Data Availability

Data is available upon request to the corresponding author.
