# Supplementary material for: Secondary-type mutations do not impact outcome in NPM1-mutated acute myeloid leukemia – implications for the European LeukemiaNet risk classification
Source: Leukemia. 2023 Sep 7;37(11):2282–5. doi: 10.1038/s41375-023-02016-6 (PMC10624615; doi:10.1038/s41375-023-02016-6)
Supplement: Supplementary file 1 — Supplements [file 41375_2023_2016_MOESM1_ESM.docx]

## **Supplements**

| **trial name** | **clinicaltrials.gov identifier** | **trial duration** | **protocol summary** |
| --- | --- | --- | --- |
| AML96 | NCT00180115 | 1996-2008 | risk-adapted postremission treatment regarding allogeneic stem cell transplantation for high-risk AML and related allogeneic and autologous stem cell transplantation for standard-risk AML, and randomization between intermediate-dose and high-dose cytarabine within the first post-remission course |
| AML2003 | NCT00180102 | 2003-2009 | early allogeneic stem cell transplantation in post-induction aplasia for high-risk AML, factorial design with four therapy arms with two factors of two stages (intensified vs. standard therapy and cytarabine vs. cytarabine + mitoxantrone + amsacrin) |
| AML60+ | NCT00180167 | 2005-2010 | Patients ≥ 60 years, mitoxantron on day 1,2,3 + cytarabine on days 1,3,5,7 vs. DA 7+3 |
| SORAML | NCT00893373 | 2011-2014 | Standard therapy + sorafenib vs. standard therapy + placebo |
| SAL bioregistry | NCT03188874 | 2010-present | Prospective registry of AML patients |
| AMLCG-1999 | NCT00266136 | 1999-2007 | double induction with HAM-HAM, multiple course G-CSF or myeloablative consolidation with Bu/Cy and autologous blood stem cell transplantation instead of maintenance vs. standard therapy |
| AMLCG-2008 | NCT01382147 | 2008-2012 | S-HAM escalated for younger patients and S-HAM basis for elderly patients vs. TAD-HAM (younger) or HAM-HAM (elderly) |

**Table S1.** Summary of clinical trial protocols and treatment regimens of previously conducted clinical trials the pooled cohort is recruited from.

| **Parameter** | **STM mutated** | **STM wildtype** | ***p*** |
| --- | --- | --- | --- |
| **n/N (%)** | 125/936 (13.4) | 811/936 (86.6) |  |
| **Age (years), median (IQR)** | 59 (49-68) | 55 (45-64) | **0.003** |
| **Sex, n (%)** |  |  | 0.081 |
| female | 63 (50.4) | 477 (48.8) |  |
| male | 62 (49.6) | 334 (41.2) |  |
| **ELN-Risk 2022, n (%)** |  |  |  |
| favorable | 75/125 (60.0) | 421/811 (51.9) | 0.102 |
| intermediate | 19/125 (15.2) | 330/811 (40.7) | **<0.001** |
| adverse | 24/125 (19.2) | 21/811 (2.6) | **<0.001** |
| missing | 7/125 (5.6) | 39/811 (4.8) |  |
| **Complex karyotype, n (%)** |  |  | 1.000 |
| No | 117/125 (93.6) | 761/811 (93.8) |  |
| Yes | 1/125 (0.8) | 11/811 (1.4) |  |
| missing | 7/125 (5.6) | 39/811 (4.8) |  |
| **Normal karyotype, n (%)** |  |  | 0.535 |
| No | 11/125 (8.8) | 89/811 (11.0) |  |
| Yes | 107/125 (85.6) | 683/811 (84.2) |  |
| missing | 7/125 (5.6) | 39/811 (4.8) |  |
| **TP53 status, n (%)** |  |  | 0.668 |
| mutated | 2/125 (1.6) | 10/811 (1.2) |  |
| unmutated | 123/125 (98.4) | 801/811 (98.8) |  |
| **Myleodysplasia-related cytogenetics** |  |  | 0.391 |
| present | 2/125 (1.6) | 30/811 (3.7) |  |
| not present | 116/125 (92.8) | 749/811 (92.4) |  |
| missing | 7/125 (5.6) | 32/811 (3.9) |  |
| **allogeneic stem cell transplantation** |  |  |  |
| in first CR | 9/125 (7.2) | 125/811 (15.4) | **0.013** |
| as salvage therapy | 13/125 (10.4) | 130/811 (16.2) | 0.110 |
| **Laboratory, median (IQR)** |  |  |  |
| WBC (10^9^/l) | 22.2 (7.1-64.1) | 39.7 (14.8-82.9) | **<0.001** |
| HB (mmol/l) | 6.6 (5.2-8.8) | 6.8 (5.5-8.6) | 0.945 |
| PLT (10^9^/l) | 46.5 (24.5-85) | 65.0 (37-112) | **<0.001** |
| BMB (%) | 80.0 (62.0-90.0) | 79.5 (61.0-90.0) | 0.694 |

**Table S2 Baseline patient characteristics with respect to secondary type mutation status.**Abbreviations: acute myeloid leukemia (AML), secondary AML (sAML), therapy-associated AML (tAML), bone marrow blasts (BMB), hemoglobin (HB), interquartile range (IQR), number (n/N), peripheral blood blasts (PBB), platelet count (PLT), secondary type mutations (STM), white blood cell count (WBC). Boldface indicates statistical significance (*p*<0.05).

| **Outcome** | **STM mut.** | **STM wt.** | **OR/HR** | ***p*** |
| --- | --- | --- | --- | --- |
| n/N (%) | 114/855 (13.3) | 741/855 (86.7) |  |  |
| CR rate, n (%) | 86/114 (75.4%) | 578/741 (78.0) | 0.87 [0.55-1.37] | 0.541 |
| RFS | 33.2 [13.9-56.5] | 26.6 [20.1-39.4] | 1.03 [0.77-1.37] | 0.859 |
| OS | 27.4 [15.2-53.3] | 32.6 [15.2-53.5] | 1.13 [0.88-1.46] | 0.325 |

**Table S3 Summary of patient outcome with respect to secondary type mutation status (STM) in *NPM1*-mutated AML excluding patients with mutated *TP53* and myelodysplasia-related cytogenetics.** Survival times are displayed in months. Square brackets show 95%-confidence intervals. Boldface indicates statistical significance (p<0.05). Abbreviations: complete remission (CR), hazard ratio (HR), mutated (mut.), number (n/N), odds ratio (OR), overall survival (OS), relapse-free-survival (RFS), wild-type (wt).


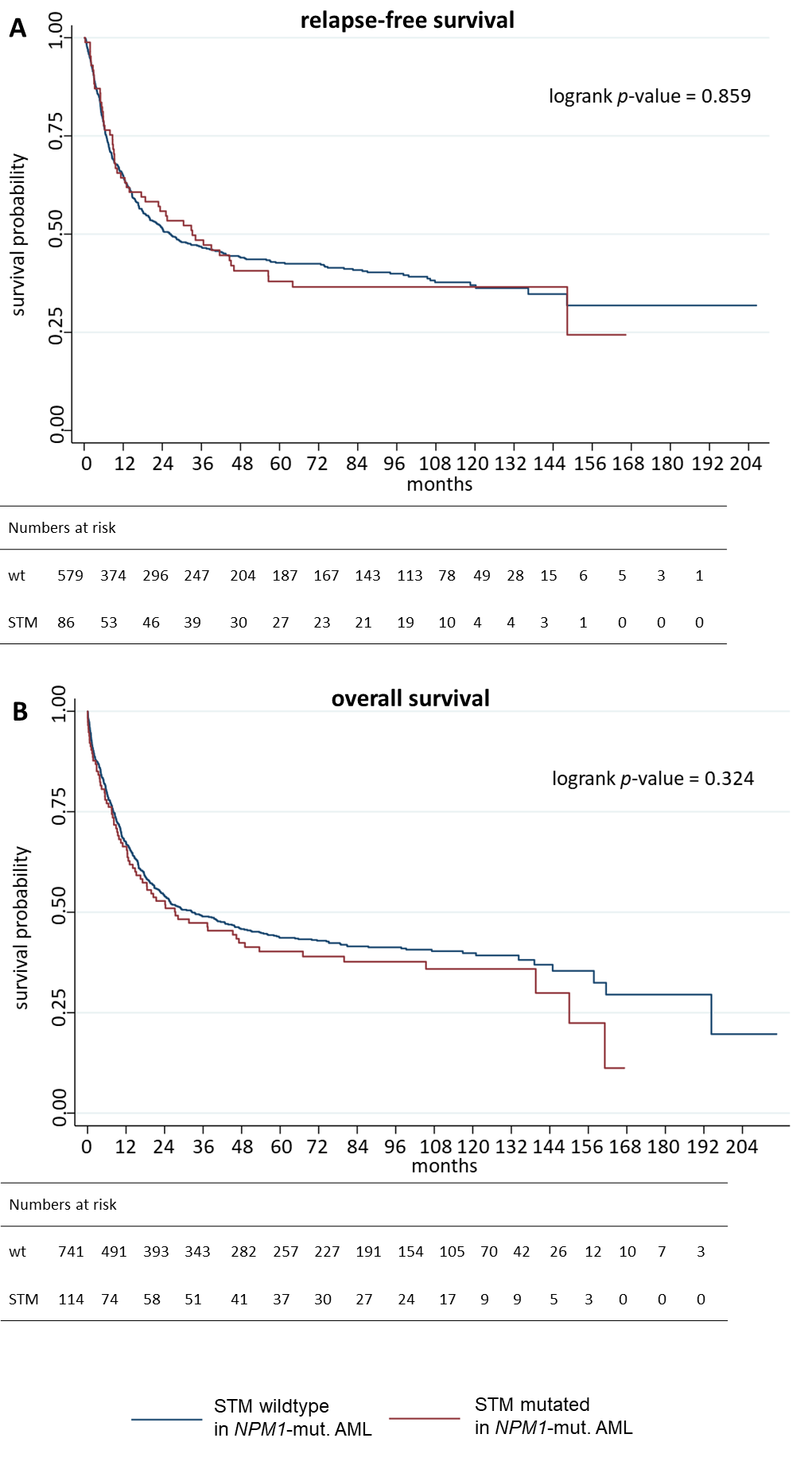


**Figure S1 Outcome of *NPM1*-mutated patients with or without co-occurring secondary type mutations (STM) excluding patients with mutated *TP53* or myelodysplasia-related cytogenetics**

| **Outcome** | ***TP53* mut.  + *NPM1* mut.** | ***TP53* wt.  + *NPM1* mut.** | **OR/HR** | ***p*** |
| --- | --- | --- | --- | --- |
| n/N (%) | 12/936 (1.3) | 924/936 (98.7) |  |  |
| CR rate, n (%) | 8/12 (66.7%) | 715/924 (77.4) | 0.58 [0.17-1.96] | 0.385 |
| RFS | 6.6 [4.5-n.r] | 25.8 [20.0-34.2] | 1.18 [0.49-2.86] | 0.707 |
| OS | 9.7 [5.0-46.9] | 29.1 [23.9-40.3] | 1.47 [0.76-2.85] | 0.249 |

**Table S4 Summary of patient outcome with respect to *TP53* mutation status in *NPM1*-mutated AML.** Survival times are displayed in months. Square brackets show 95%-confidence intervals. Boldface indicates statistical significance (p<0.05). Abbreviations: complete remission (CR), hazard ratio (HR), mutated (mut.), number (n/N), not reached (n.r.), odds ratio (OR), overall survival (OS), relapse-free-survival (RFS), wild-type (wt).


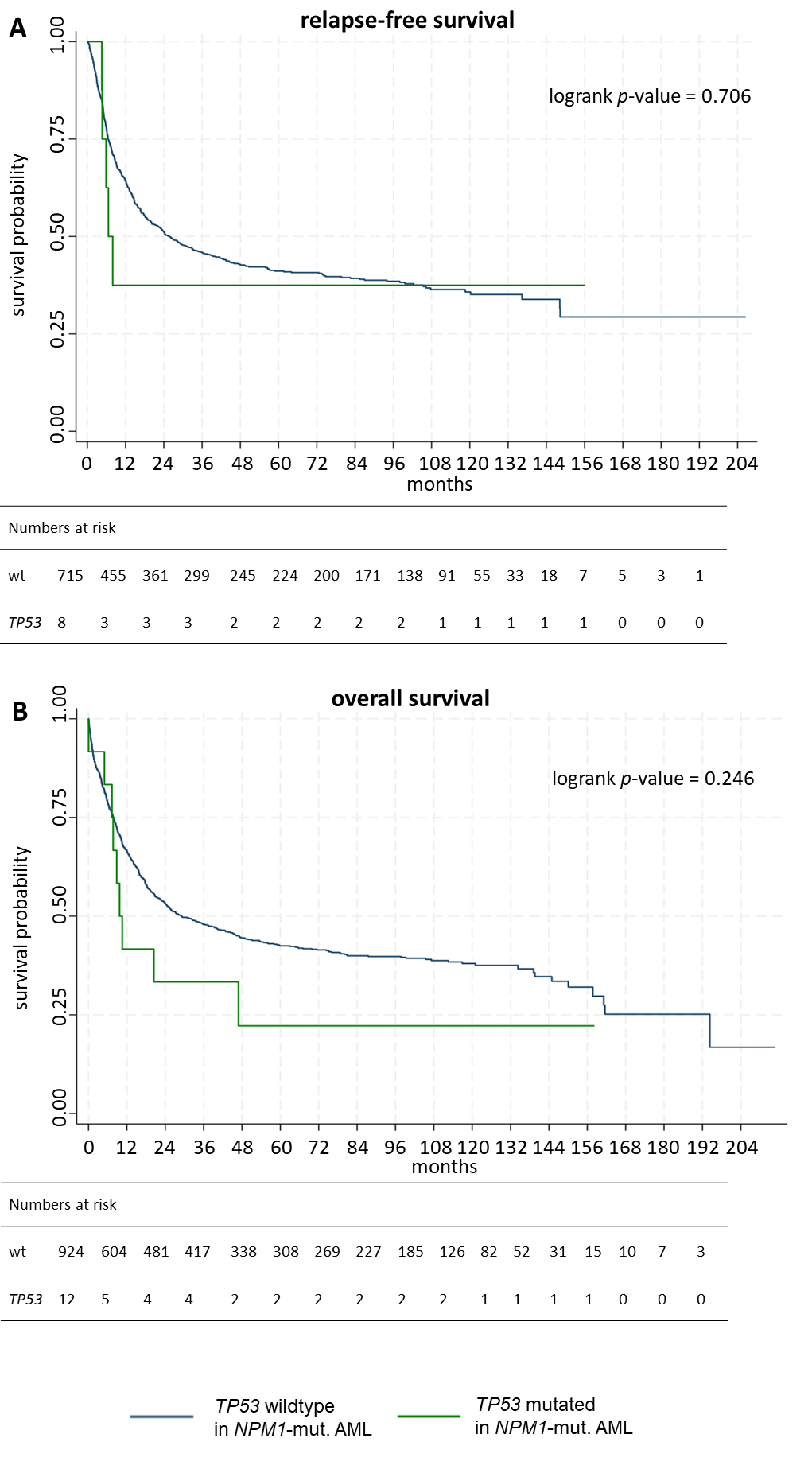


**Figure S2 Outcome of *NPM1*-mutated patients with or without co-occurring *TP53* mutations**

| **Outcome** | ***NPM1* mut. with myelodysplasia-rel. cytogenetics** | ***NPM1* mut. without myelodysplasia-rel. cytogenetics** | | **OR/HR** | ***p*** |
| --- | --- | --- | --- | --- | --- |
| n/N (%) | 17/936 (1.8) | 919/936 (98.2) |  | |  |
| CR rate, n (%) | 14/17 (82.4%) | 709/919 (77.1) | 1.38 [0.39-4.86] | | 0.614 |
| RFS | 13.2 [4.2-102.4] | 25.5 [19.7-34.6] | 1.48 [0.81-2.69] | | 0.200 |
| OS | 35.7 [7.5-n.r] | 28.2 [23.5-39.9] | 1.07 [0.59-1.94] | | 0.824 |

**Table S5 Summary of patient outcome with respect to presence or absence of co-occurring myelodysplasia-related cytogenetics in *NPM1*-mutated AML.** Survival times are displayed in months. Square brackets show 95%-confidence intervals. Boldface indicates statistical significance (p<0.05). Abbreviations: complete remission (CR), hazard ratio (HR), mutated (mut.), number (n/N), not reached (n.r.), odds ratio (OR), overall survival (OS), relapse-free-survival (RFS), wild-type (wt).


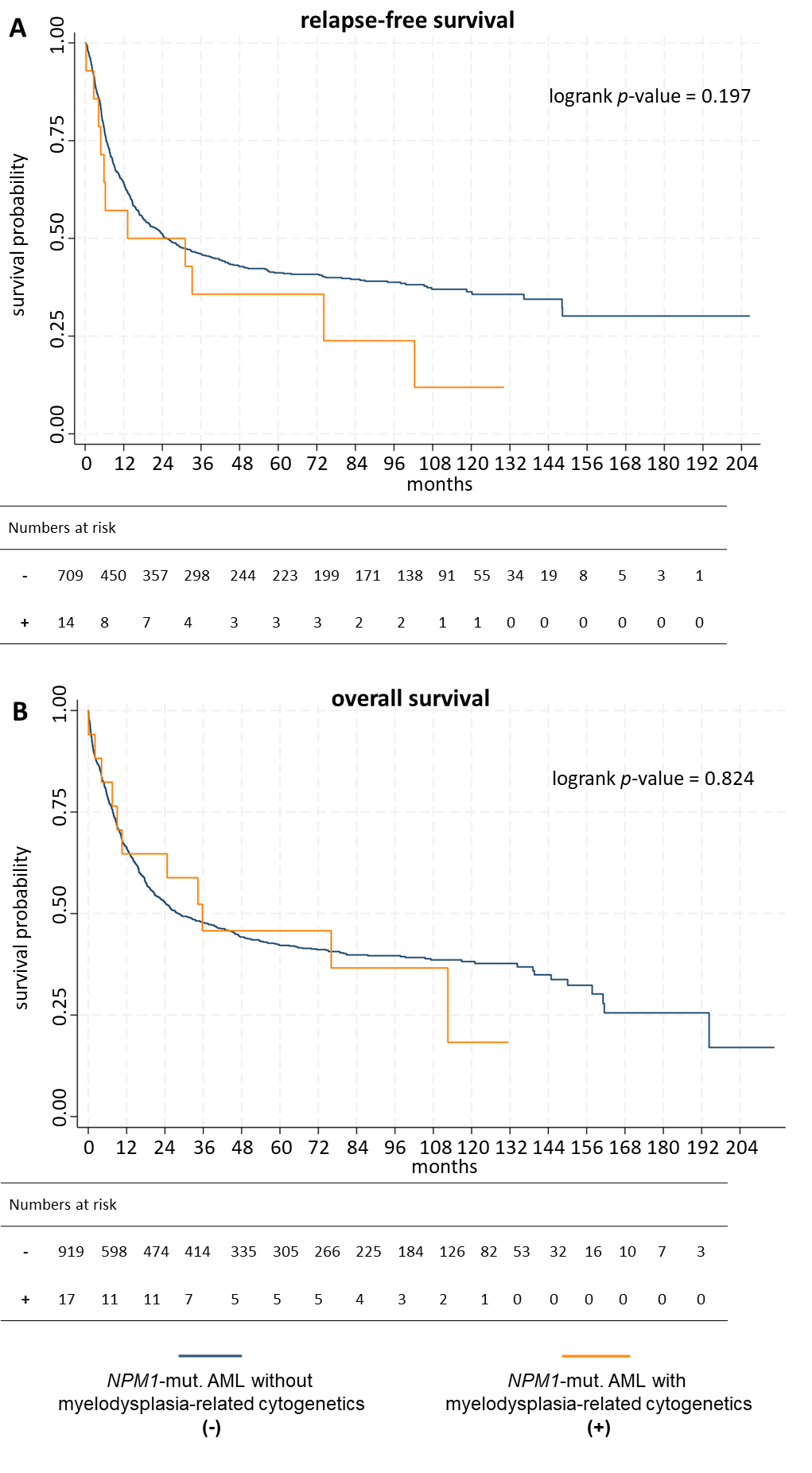


**Figure S3 Outcome of *NPM1*-mutated patients with or without co-occurring myelodysplasia-related cytogenetics**

| **Outcome** | **STM mut.** | **STM wt.** | **OR/HR** | ***p*** |
| --- | --- | --- | --- | --- |
| n/N (%) | 75/495 (15.2) | 420/495 (84.8) |  |  |
| CR rate, n (%) | 53/75 (70.7%) | 336/420 (80.0) | 0.60 [0.35-1.05] | 0.072 |
| RFS | 46.0 [25.1-n.r.] | 49.7 [32.9-107.7] | 1.08 [0.73-1.59] | 0.702 |
| OS | 45.3 [19.8-105.4] | 59.8 [43.8-144.9] | 1.31 [0.96-1.81] | 0.092 |

**Table S6 Summary of patient outcome with respect to secondary type mutation status in *NPM1*-mutated AML within ELN2022 favorable risk.** Survival times are displayed in months. Square brackets show 95%-confidence intervals. Boldface indicates statistical significance (p<0.05). Abbreviations: complete remission (CR), hazard ratio (HR), mutated (mut.), number (n/N), not reached (n.r.), odds ratio (OR), overall survival (OS), relapse-free-survival (RFS), wild-type (wt).

**
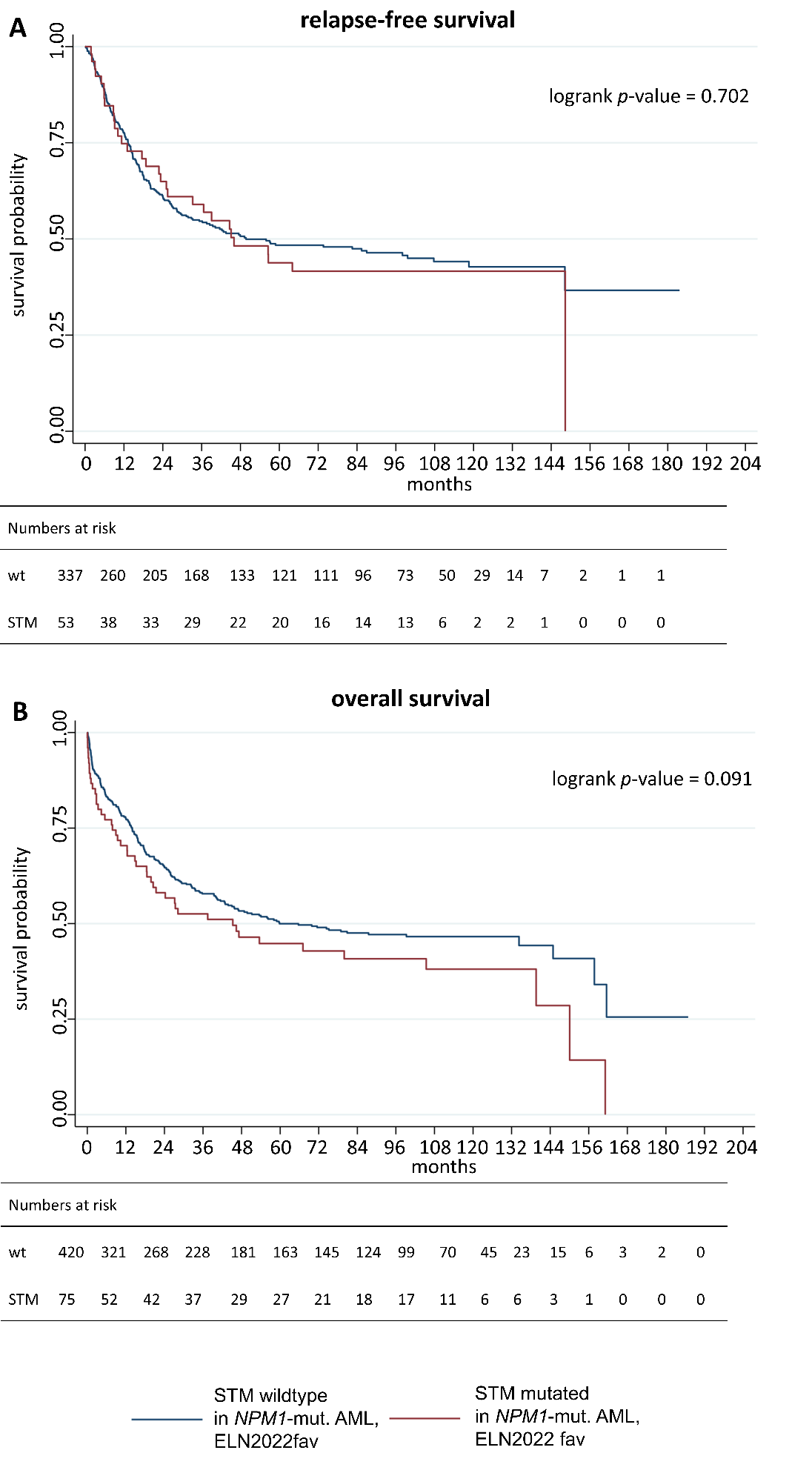
**

**Figure S4 Outcome of *NPM1*-mutated patients with or without co-occurring secondary type mutations within the ELN2022 favorable risk group**

| **Outcome** | **STM mut.** | **STM wt.** | **OR/HR** | ***p*** |
| --- | --- | --- | --- | --- |
| n/N (%) | 103/659 (15.6) | 556/659 (84.4) |  |  |
| CR rate, n (%) | 73/103 (70.9%) | 397/556 (71.4) | 0.97 [0.61-1.55] | 0.913 |
| RFS | 32.9 [11.3-45.1] | 32.7 [20.9-73.9] | 1.17 [0.85-1.61] | 0.334 |
| OS | 20.4 [10.4-37.5] | 17.8 [14.3-22.8] | 1.07 [0.83-1.38] | 0.621 |

**Table S7 Summary of patient outcome with respect to secondary type mutation status in *NPM1*-mutated AML excluding patients who received allogeneic stem cell transplantation.** Survival times are displayed in months. Square brackets show 95%-confidence intervals. Boldface indicates statistical significance (p<0.05). Abbreviations: complete remission (CR), hazard ratio (HR), mutated (mut.), number (n/N), odds ratio (OR), overall survival (OS), relapse-free-survival (RFS), wild-type (wt).


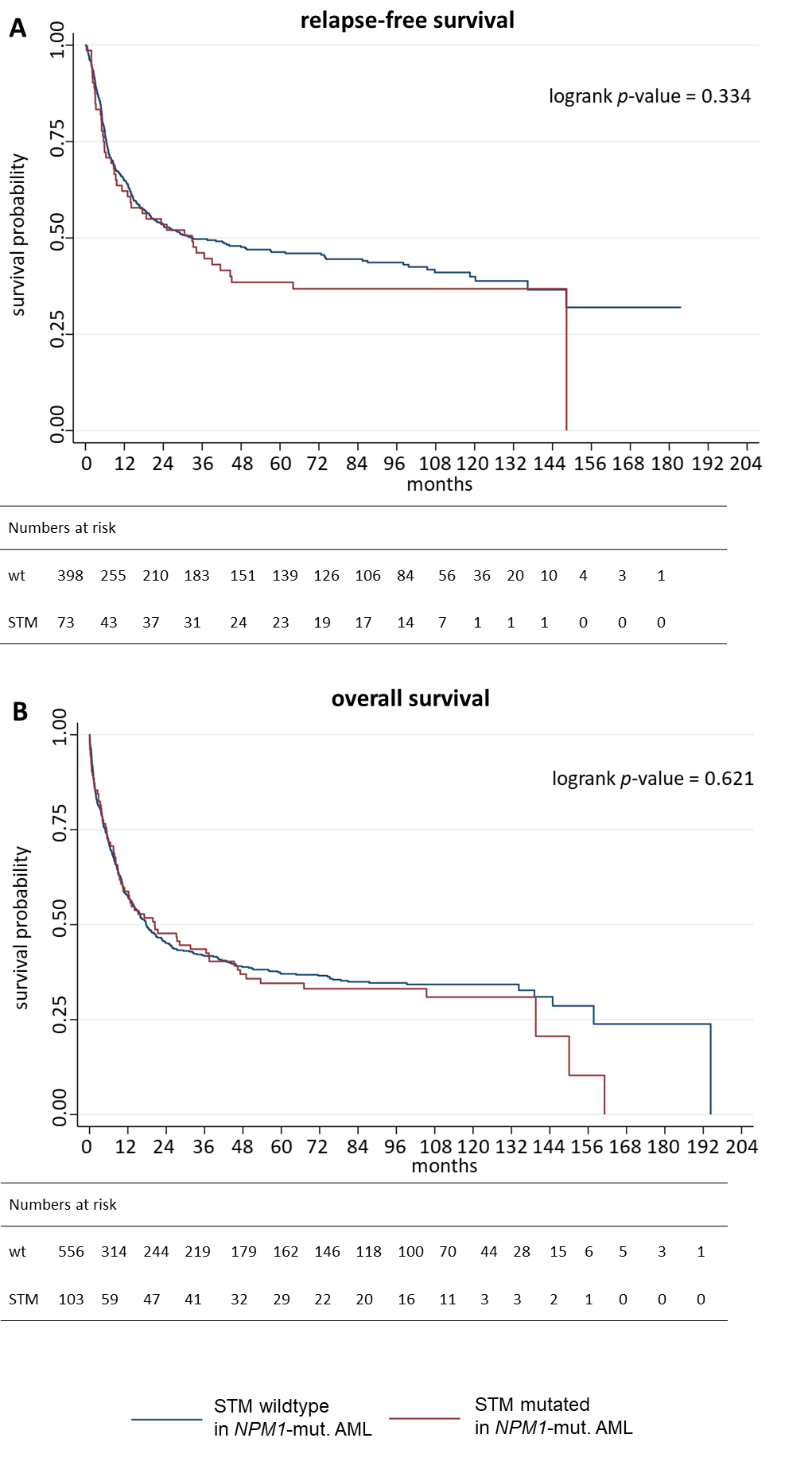


**Figure S5 Outcome of *NPM1*-mutated patients with or without co-occurring secondary type mutations excluding patients who received allogeneic hematopoietic stem cell transplantation**
